# Self-Assembling Peptides and Carbon Nanomaterials Join Forces for Innovative Biomedical Applications

**DOI:** 10.3390/molecules26134084

**Published:** 2021-07-04

**Authors:** Petr Rozhin, Costas Charitidis, Silvia Marchesan

**Affiliations:** 1 Chemical and Pharmaceutical Sciences Department, University of Trieste, 34127 Trieste, Italy; petr.rozhin@phd.units.it; 2 School of Chemical Engineering, National Technical University of Athens, Iroon Polytechneiou 9, Zografou, 157 80 Athens, Greece; charitidis@chemeng.ntua.gr; 3 INSTM, Unit of Trieste, 34127 Trieste, Italy

**Keywords:** self-assembly, peptides, amyloids, carbon dots, graphene, carbon nanotubes, fullerene, nanostructures, biomaterials, hydrogels

## Abstract

Self-assembling peptides and carbon nanomaterials have attracted great interest for their respective potential to bring innovation in the biomedical field. Combination of these two types of building blocks is not trivial in light of their very different physico-chemical properties, yet great progress has been made over the years at the interface between these two research areas. This concise review will analyze the latest developments at the forefront of research that combines self-assembling peptides with carbon nanostructures for biological use. Applications span from tissue regeneration, to biosensing and imaging, and bioelectronics.

## 1. Introduction

### 1.1. Self-Assembling Peptides

Supramolecular systems based on self-assembling peptides have become a very popular topic of investigation for multidisciplinary research [1,2]. There are many reasons that render these building blocks very attractive. First of all, amino acids feature a very large chemical diversity that has been further extended with the introduction of numerous non-natural derivatives; thus, making it possible to virtually encode any kind of functional group into a peptide [3,4]. Secondly, their preparation can be conveniently attained by a variety of methods, with the modular solid-phase synthesis standing out for simplicity, also for the non-expert, as well as ease of purification [5]. Thirdly, short peptide sequences can exert part of the functionalities displayed by complex proteins, thus, allowing for their biomimicry using much simpler, and often more robust, molecules [6]. Fourth, the choice of encoding a desired function within a supramolecular architecture opens the door to smart systems that respond to external stimuli with assembly/disassembly cycles, with consequent on/off switching of the encoded function. This bioinspired feature is very attractive for biomedical applications [7,8], spanning from the development of smart antimicrobials [9], to advanced vehicles for therapeutics [8,10] and protein [11] delivery. The additional advantages of inherent biodegradability and biocompatibility render self-assembling peptides ideal building blocks also for vaccine development [12], imaging [13,14], biomaterials design [15], wound healing [16], and cancer therapy [17,18].

**Figure 1:**
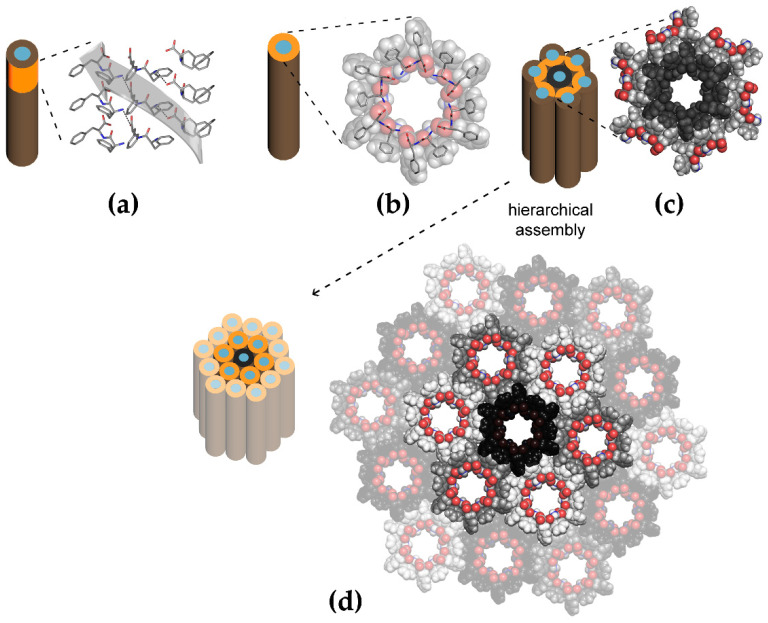
Schematic representation of amyloid-derived diphenylalanine self-assembled nanotubes from single-crystal XRD data [41]. (**a**) Side-view of the dipeptide stacks along the nanotube, (**b**–**d**) top-view of the (**b**) individual nanotubes, and (**c**,**d**) their progressive bundling into microtubes. Adapted from [42] under a Creative Commons license (https://creativecommons.org/licenses/by/4.0/, accessed on 8 June 2021).

Self-assembling peptides are often derived from amyloids, which have been recognized as important biotechnological tools, besides their relevance in physiological and pathological contexts [19]. Amyloid proteins and peptides display a typical cross-β structure with molecular backbones being perpendicular to the long axis of the fibrils and interacting with each other through ordered hydrogen bonding, while amino acid side-chains typically engage in steric zippers of a hydrophobic nature [20]. Among the amyloid-derived short peptides, the most popular building block used for self-assembly is diphenylalanine [21]. This minimalistic building block with a strong self-association propensity into nanotubes (Figure 1) was identified through a reductionist approach from the amyloid β-peptide sequence [22]. Since then, a large variety of derived motifs have been used to design self-organizing derivatives [23]. Several reviews have appeared in the last year on self-assembling short peptides for biomedical applications [24,25], especially drug delivery [26,27] and tissue engineering [28], also owing to their ability to mimic the extracellular matrix [29]. More specifically, their use has been reviewed as microgels [30], antimicrobials [31], for angiogenesis [32], in gene therapy [33], to treat metabolic syndromes [34] and gastrointestinal diseases [35], to regenerate bone [36] and conductive tissues [37] such as nerves [38], to develop bioelectronics [39] and vaccines [40]. Therefore, in this review we will not analyze in detail these highly promising systems.

However, peptide-based systems display also certain limitations; therefore, the research has been very active to develop composite [43] or hybrid [44] nanostructures and materials with additional components to ameliorate their features and introduce new properties. Numerous examples have been reported to date and with a great variety of chemical components, such as polymers [45], polysaccharides [46], nucleic acids [47], inorganic nanoparticles [48,49], polyoxometalates [50], metal-organic cages [51], and more.

### 1.2. Carbon Nanomaterials

**Figure 2:**
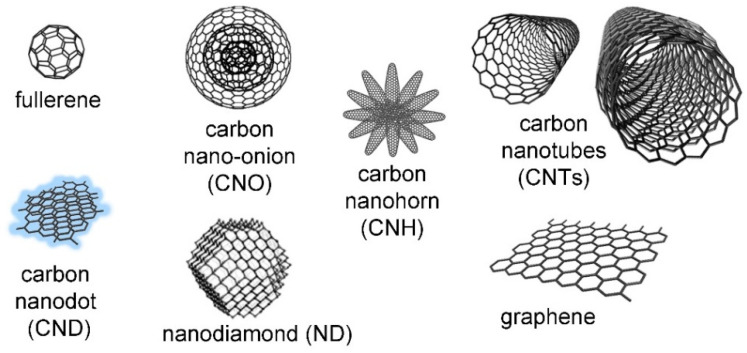
Carbon nanomaterials (not to scale), reproduced from [61] under a Creative Commons license (https://creativecommons.org/licenses/by/4.0/, accessed on 8 June 2021). The nano-onion is reproduced from [62], copyright ©1995 with permission from Elsevier.

A specific type of additives that deserves a detailed discussion comprises the family of carbon nanostructures (Figure 2). They all share the common feature of being composed by carbon atoms, which are, in the majority of cases, sp^2^ hybridized and inter-connected in a honeycomb lattice [52]. Nevertheless, they can be extremely diverse both in terms of structure and morphology, thus, in their reactivity and physico-chemical properties. They comprise 0D fullerenes [53], nano-onions [54], and luminescent nanodots [55]. One-dimensional carbon nanotubes (CNTs) [56] feature a tubular morphology, while graphene-based materials [57] can be considered 2D. Other examples include clusters of nanocones termed nanohorns (CNHs) [58] and nanodiamonds (NDs), which feature a large amount of sp^3^-hybridized carbon atoms [59] and are promising for various biomedical uses [60].

All these diverse carbon nanostructures have been widely studied, yet it is not straightforward to predict which is the ideal candidate based on the type of intended application. This challenge is amplified in biologically relevant contexts, which are characterized by a high-level of chemical complexity [63,64,65,66,67]. To this end, it is key to study their different ability to interact with biomolecules and develop a protein corona on their surface [68] that will influence their ability to elicit an immune response [69,70], as well as their biodegradation [71,72] and biodistribution [73].

Despite their morphological diversity, they do share certain common features, including a low density, high mechanical strength, good electronic conductivity, and they provide the opportunity to further tailor their properties upon chemical functionalization [74]. In addition, carbon nanostructures display a high-surface area and hydrophobic nature that can be exploited to non-covalently load high levels of bioactive molecules, as widely applied in drug delivery [75]. As a result, they have attracted great interest for their innovative potential in areas of unmet clinical need [74,76], such as oncology [77], infections [78], and tissue engineering [79], especially for conductive nerve [80,81] and cardiac [82,83] tissues, but also bone [84]. Their potential applications in sensing [85] and, generally, in clinical applications [86] are also widely studied. 

Nevertheless, today there are still concerns regarding possible side-effects from the use of carbon nanomaterials [87,88], and their great heterogeneity renders the task even more challenging to assess [89]. A useful approach to address this issue is to develop unified standards for their classification, which is a complex task that is being tackled by various committees [90]. A general framework for a reliable risk assessment approach to develop nanotechnology responsibly is a common objective for societal welfare [91]. Innovative ways for more efficient data management [92] and inclusion of modern in silico methods, such as machine learning to make the most out of large datasets, hold the key to tackle this type of unresolved issues [93].

### 1.3. Combination of Self-Assembling Peptides and Carbon Nanomaterials

The combination of self-assembling peptides with carbon nanomaterials can provide a very interesting opportunity to innovate in the biomedical sector, given their highly diverse properties. On one hand, peptides are ideal components for biomimicry, to exert bioactivity [94] and favorably interact with water [95]. On the other, carbon nanomaterials’ conductivity and mechanical resilience can provide additional properties to peptide-based systems [96]. Furthermore, synergy can be created by leveraging on their very different chemical nature. For instance, one of the main limitations of carbon nanomaterials is their hydrophobic nature and tendency to aggregate that may be alleviated through chemical functionalization [97]. Alternatively, the amphiphilic nature of self-assembling peptides can be convenient to enhance carbon nanomaterials’ dispersibility in water [66,98,99]. Ordered oligoglycines, for instance, were shown to coat oxidized CNTs or GO thanks to non-covalent interactions between the carboxylic acid groups on the carbon nanomaterials and the N-terminus of the peptides, with potential applications in conductive tissue regeneration [100]. 

In addition, peptides can be used for active targeting [101], cell penetration [102], to direct biodegradation [103], as fluorescent probes [104], and so on. However, the very different chemical nature of these two types of components renders their combination quite a challenge [105], and as a result this area of research is still underexplored. Furthermore, in the case of self-assembling peptides, it is possible that the presence of carbon nanomaterials can have detrimental effects on their supramolecular behavior. This represents a further challenge for exploiting their interaction in useful ways. In certain cases, the inhibitory effect can be useful, for instance to target fibrillation of pathological amyloids [67,106]. In this concise review, we will, thus, delineate how self-assembling peptides and carbon nanomaterials can indeed join forces to innovate in the biomedical sector, and we will focus on the very latest research efforts of the last few years in this inspiring area.

## 2. Research on the Interaction between Self-Assembling Peptides and Nanocarbons

Despite the fact that carbon nanomaterials are a large group that comprises many different types of structures and morphologies as shown in Figure 2, the majority of recent studies that combine them with self-assembling peptides concerns graphene-based materials, and, to a lesser extent, CNTs and carbon dots. Over the years, research pertaining to the design of nanostructures and self-assembling building blocks has achieved great advances, so that we have witnessed a progressive shift from 2D [107] to 3D-architectural complexity [108]. This section will describe the latest developments in this area, dividing the most recent reports based on the type of nanocarbon, and following a progression from 0D materials (i.e., fullerenes and carbon dots) to 1D components (i.e., CNTs), to the 2D graphene-based materials (e.g., graphene, graphene oxide or GO, and reduced graphene oxide or rGO). In some cases, more than one carbon nanostructure has been combined with self-assembling peptides in multi-component systems [109,110,111]. Recent examples are reported in Table 1, following the same progressive order from 0D to 1D and 2D carbon nanomaterials.

### 2.1. Fullerenes 

Fullerenes are hollow, spherically shaped structures with a very interesting affinity for electrons and their transport that renders them attractive for applications in energy and photocatalysis [142], as well as photodynamic therapy [143]. In medicine, fullerenes have been widely studied as antioxidants [144,145], antimicrobials [146], and vehicles for drug delivery [147,148]. Fullerenes, and in particular C_60_, have been studied also for their ability to interfere with amyloid aggregation [106]. However, fullerenes’ poor solubility in water is a limiting factor for their biological application, for which more hydrophilic derivatives are preferred, such as fullerenols that carry hydroxyl groups [149]. A recent study compared in silico the ability of C_60_, C_60_(OH)_6_ and C_60_(OH)_12_ to inhibit the fibrillation of amyloid beta (Aβ) peptides, concluding that C_60_(OH)_6_ led to the best inhibitory performance thanks to its balanced amphipathic character, which allowed hydrophobic interactions as well as hydrogen bonding with the peptide backbone [112]. Another hydrophilic C_60_ derivative, 1,2-(dimethoxymethano)fullerene, was studied for the same purpose as it demonstrated to be a fibrillation inhibitor for Aβ in silico [113].

**Figure 3:**
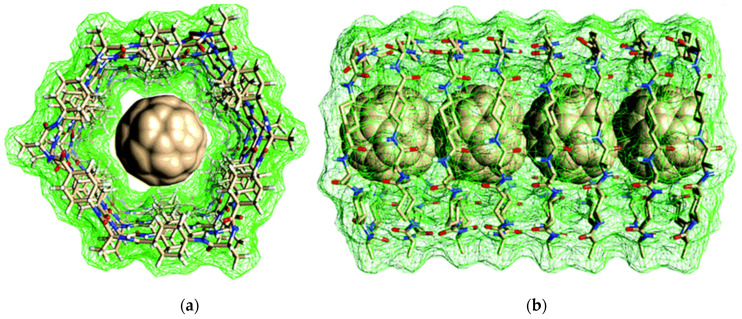
(**a**) Top-view and (**b**) side-view of a computer-assisted model of C_60_ encapsulated in the inner cavity of supramolecular nanotubes arising from the stacking of cyclopeptides. Adapted from [114] published by the Royal Society of Chemistry under a Creative Commons license (https://creativecommons.org/licenses/by/4.0/, accessed on 8 June 2021).

Hydrophobic interactions have been used also to allow the encapsulation of C_60_ within supramolecular peptide nanotubes formed through stacking of cyclopeptide derivatives (Figure 3). This type of hybrid nanostructure was envisaged for potential use in drug delivery or electronic applications. C_70_ could not be efficiently encapsulated with the same strategy presumably due to its bigger size [114]. Conversely, when self-assembling peptide cavitands were used for fullerene encapsulation, both C_60_ or C_70_ could be suitable guests [150].

Peptides can indeed be very convenient to enhance fullerenes’ solubility in water and direct their supramolecular behavior, whilst maintaining their electronic properties and resulting antioxidant activity, as demonstrated through covalent conjugation [151,152]. Alternatively, a non-covalent approach can be envisaged, for instance by embedding fullerenes within the hydrogel matrix formed by self-assembling peptides; the resulting hybrid material could be envisaged for photodynamic antibacterial therapy, because of the fullerenes’ well-established electronic properties with the additional benefit of the injectable matrix formed due to the presence of the peptide [115]. 

### 2.2. Carbon Dots

Carbon dots are a more recent type of carbon nanomaterials that can be conveniently prepared through top-down or bottom-up approaches [153], also starting from small molecules in a green manner [61], and have very promising photoluminescence and biocompatibility profiles for applications in medicine [154,155]. They are widely studied, especially for biosensing applications, given their tunable optoelectronic properties [156].

**Figure 4:**
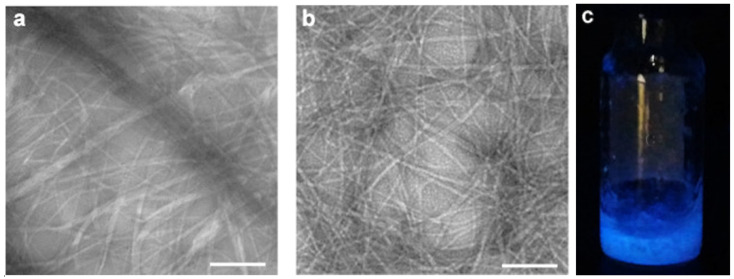
Supramolecular hydrogels from a self-assembling tripeptide (**a**) without or (**b**,**c**) with carbon dots. (**a**,**b**) Transmission electron microscopy (TEM) micrographs reveal that presence of the dots yields a homogeneous population of peptide fibrils (**b**), as it inhibits the hierarchical bundling of fibrils into heterogeneously sized fibers that occurs with the peptide alone (**a**). The hybrid gel is luminescent as seen under a UV light (**c**). Scale bar = 200 nm. Adapted from [116].

Carbon dots have demonstrated in some cases to have detrimental effects on the self-assembly of amyloid peptides, although it is possible to turn this phenomenon into an advantage, for instance by hindering hierarchical bundling of amyloids and stabilizing instead the formation of individual fibrils to attain a more homogeneous and luminescent hydrogel (Figure 4) [116].

**Figure 5:**
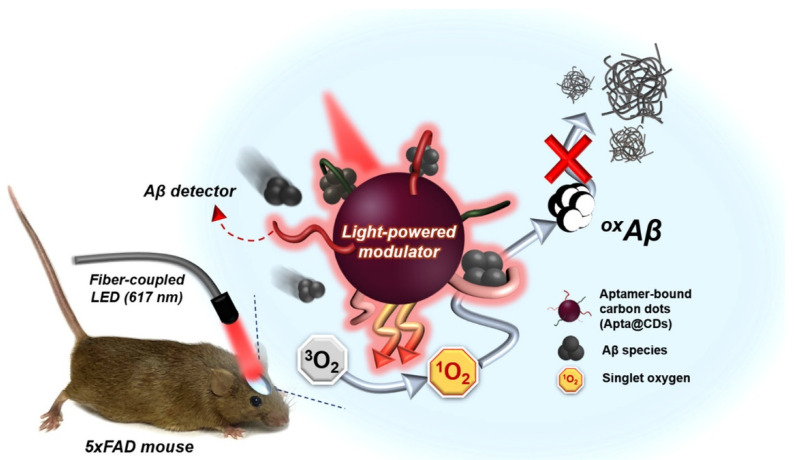
Carbon dot-mediated photo-modulated Aβ aggregation inhibition in vivo. The aptamer-functionalized carbon dots (Apta@CDs) capture Aβ species in neurophysiological conditions, and, under red-light irradiation, they generate ^1^O_2_ to chemically and irreversibly denature Aβ peptides for effective inhibition of neurotoxic amyloid aggregation. Reproduced with permission from Chung, Y. J. et al., *ACS Nano* [118], © 2021 American Chemical Society.

Alternatively, they can be used to inhibit pathological amyloid aggregation, although to this end, GO was shown to be more effective [67], yet clearly the performance could depend on a plethora of physicochemical properties of the specific nanomaterials of choice, including not only the size and morphology, but also the type and density of functional groups as well as the experimental conditions. For instance, dost can be effective for this application, as shown for nitrogen-doped carbon dots displaying intense photoluminescence at 550 nm, when excited at 420 nm, that originated from the n → π* transition of N-groups. The amine and imine groups on their surface effectively chelated Cu divalent cations, thus, preventing Cu(II)-induced Aβ aggregation as well as the amyloid aggregation in the absence of copper. Moreover, irradiation of the dots with a blue LED generated radical oxygen species that oxidized the amyloid peptide, thus, providing yet another means to inhibit its aggregation [117]. This type of approach could be further improved through the carbon dots’ functionalization with an aptamer for specific targeting, and through preparation from different precursors to attain red-light responsiveness, for better tissue penetration in vivo (Figure 5) [118]. 

Graphene carbon dots have been proposed as imaging agents to monitor amyloid fibrillation. One advantage is that they display tunable emission that depends on the excitation wavelength. In one study, excitation at 400 nm was shown to be particularly favorable to yield intense emission at 500 nm that was quenched by the presence of the amyloid β peptide monomer, whilst not by its fibrils. The authors ascribed the phenomenon to the ability of amyloid β, only in the monomer state, to favorably interact with the graphene quantum dots for an excited-state electron energy transfer that resulted in luminescence quenching [119]. However, it is worth noting that amyloids present intrinsic fluorescence that is independent from the peptide sequence [157]. It has been ascribed to the refolding from helical to β-sheet structures and it covers the whole visible region (400–650 nm), finding potential applications for bioimaging in vivo [14].

Amyloids occur in a variety of pathologically-relevant contexts that go well beyond neurodegenerative diseases, and are recurrent also in prokaryotes [158]. For instance, *Staphylococcus aureus* mature biofilms are rich in amyloids, and graphene quantum dots were shown to effectively disaggregate them, possibly acting on the nuclei and altering their structure; thus, hindering further elongation into fibrils [120].

Alternatively, amyloid peptides can be bound to carbon dots to attain nanostructured materials for theranostics. As an example, the peptide sequence RGDAEAKAEAKYWYAFAEAKAEAKRGD was chosen as it contained (1) the RGD bioactive motif to target cancer cells, (2) the YWYAF motif for selective binding onto graphenic surfaces, and (3) the EAK motif for self-assembly into nanofibers. In this manner, the peptide elongated nanostructures bound to graphene quantum dots that were probed in vitro for the targeting and imaging of cancel cells [121].

Finally, carbon dots have been combined with GO and peptide nanofibers for the biosensing of hydrogen peroxide. In this case, the peptide sequence was designed to provide aromatic binding sites for both the dots and GO. This approach served to provide further stability to the ternary system, as well as an additional layer to hamper GO aggregation and favor the adsorption of electrolytes for electrochemical sensing and the diffusion of reagents [111]. 

### 2.3. Carbon Nanotubes (CNTs)

Carbon nanotubes feature a tubular morphology and can display one, two, or more graphitic walls [159]. Depending on how the graphene sheet is rolled into the tubular structure, different chirality and electronic properties arise [160]. High-purity sorted CNTs find a myriad of innovative applications, especially in sensing [161], nanoelectronics [162], and even implantable biosensors [163].

Interestingly, CNTs can be spun into macroscopic fibers with various degrees of CNT alignment, hence, varying electromechanical properties [164]. Their extraordinary properties in terms of conductivity, low density, high tensile strength, and so on, rendered them very attractive components for the development of innovative flexible, and even wearable, electronics [165]. Furthermore, convenient and green protocols have been developed to functionalize CNT fibers in the gas phase, with virtually no waste production and the possibility to fine-tune their hydrophilicity for use in water or ionic liquids, as needed for the intended application [166].

The development of biohybrids based on self-assembling biomolecules and CNTs is gaining momentum [167]. In particular, the similar anisotropic morphology of CNTs and peptide fibrils can favor the formation of hybrid materials, whereby the two components are intimately connected, and the resulting hydrogels display enhanced mechanical properties relative to the systems without CNTs [66,98]. Furthermore, CNTs’ dispersibility can be enhanced through non-covalent coating by self-assembling peptides that typically display an amphipathic nature [168]. Sometimes new properties, such as a self-healing behavior, can emerge from such favorable interactions [66]. The binding of amyloid peptides onto the CNTs’ surface is governed by several factors, including CNT curvature as shown by molecular dynamics’ studies [169]. The interactions between aromatic amino acids, such as tryptophan and phenylalanine, which are recurrent residues in self-assembling peptides, have been studied by molecular dynamics for their ability to yield stable supramolecular hybrid materials [124]. Alternatively, self-assembling peptides can also be covalently anchored onto the CNT’s surface, in this manner, upon the addition of free peptides, supramolecular dendritic structures were obtained, for potential applications in tissue engineering [123].

**Figure 6:**
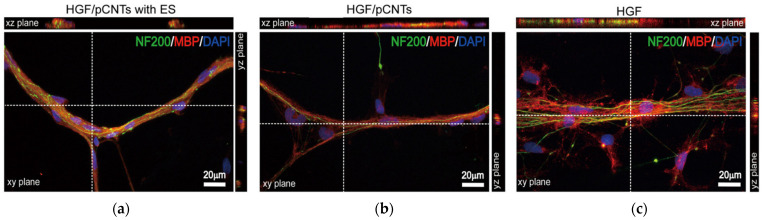
Axon myelination after a 30-day neuron cultivation under different conditions: (**a**) self-assembling peptide (HGF), pristine CNTs (pCNTs), and electrical stimulation (ES); (**b**) HGF and pristine CNTs; (**c**) HGF alone. Neurons are stained in green (NF200), myelin in red (MBP), and cell nuclei in blue (DAPI). Adapted with permission from He, L. et al., *ACS Appl. Mater. Interfaces* [122], copyright © 2021, American Chemical Society.

There are several potential applications for these systems, including smart materials that release their cargo upon application of specific stimuli, such as near-infrared light irradiation that exploits CNT photo-thermal energy conversion [125]. Other uses of this type of materials include tissue regeneration, as shown on peripheral nerves, with promising levels of myelination when coupled with electrical stimuli (Figure 6) [122]. Alternatively, aggregation-induced emission of self-assembling peptide derivatives that also act as CNT dispersants could find potential applications in sensing [99], especially if suitable conjugation strategies are adopted to maximize the performance of the resulting systems for optical detection [170]. Furthermore, CNTs have been proposed for photoacoustic imaging in vivo, which could be applied to tumor tissues through their conjugation with targeting moieties, such as cyclic Arg-Gly-Asp to target integrin proteins that were overexpressed by cancer cells [171].

### 2.4. Graphene-Based Materials

Graphene-based materials come in different form and size and their classification has been reviewed [172]. Graphene’s unique physicochemical properties, high conductivity and tensile strength, and low density have attracted great interest for biological applications [173,174]. As can be seen from Table 1, graphene oxide is possibly the most studied type of derivative to generate biomaterials scaffold, in light of its higher dispersibility in water relative to graphene. Conversely, for biosensing and bioelectronics applications, graphene is preferred for its superior electron conductivity.

De novo design of peptide sequences for optimized adsorption and self-assembly onto the graphene’s surface is a challenging task, since the binding energy is determined by cooperative effects that are not simply the sum of each amino acid contribution [175]. Compared to polyaromatic units, such as pyrene that can engage in efficient and ordered π–π stacking, peptides display weaker binding onto graphene, yet they are being studied for their ability to self-organize onto graphene and modify its electronic properties for sensing applications [128]. In particular, self-assembled diphenylalanine nanotubes are widely investigated and can form onto the surface of graphene, despite the fact that binding of the peptides onto the carbon nanomaterial’s surface does alter the hydrogen bonding pattern of the assemblies [126]. When self-assembling peptide gelators and graphene oxide are successfully combined together, the mechanical properties of the resulting materials are improved [66,138].

#### 2.4.1. Sensing Applications

**Figure 7:**
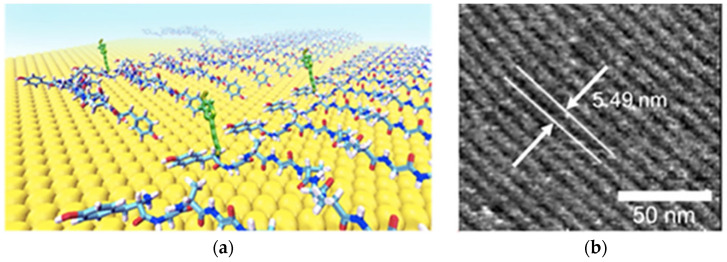
(**a**) Self-assembling peptides based on glycine–alanine repeating units form hydrogen-bonded ordered β-sheets to coat substrates (yellow) for biosensing, thanks to the inclusion of a biotin probe (green). (**b**) Atomic force microscopy shows the peptide-ordered arrays. Reproduced with permission from Li, P. et al., *ACS Appl. Mater. Interfaces* [129], copyright © 2021 American Chemical Society.

Glycine–alanine repeating units proved useful in the design of self-assembling peptides that could form stable coatings onto exfoliated graphite for biosensing applications, as demonstrated through inclusion of biotin for streptavidin detection (Figure 7) [129]. In another work, for the same purpose, the peptide sequence IMVTESSDYSSY was used for its ability to bind to graphenic substrates forming a self-assembled monolayer, and was mutated to display biotin [127]. However, typically these are proof-of-concept studies, while the most studied self-assembling peptide for more advanced sensing applications is diphenylalanine, which yields nanotubes, as previously shown in Figure 1. For instance, aligned semiconducting peptide nanotube–graphene oxide composites allowed to enhance the surface-enhanced Raman spectroscopy (SERS) relative to GO alone, allowing the nanomolar detection of glucose and nucleobases [131].

**Figure 8:**
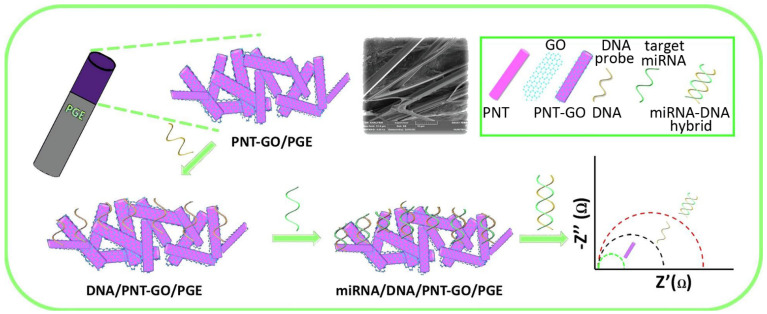
MicroRNA (miRNA-192) biosensor based on a pencil graphite electrode (PGE) coated with self-assembled diphenylalanine nanotubes (PNTs) and GO, onto which the addition of a single-stranded DNA probe allowed for hybridization with its complementary target miRNA sequence, and detection through changes in impedance. Adapted from [176] © 2021 with permission from Elsevier.

GO was also combined with diphenylalanine-peptide nanotubes to develop a sensor, in this case using a graphite electrode, due to the conductivity of the components. Subsequent inclusion of a single-stranded DNA sequence complementary to a target microRNA allowed for its detection through changes in impedance in the biosensor (Figure 8) with a remarkably wide linear range (10 fM–1.0 nM) and low sensing limit (8 fM) [176]. This study was designed for the quantification of microRNA-192, which is a useful target for oncological studies, as it is downregulated in cancer cells, of which it inhibits proliferation, migration and invasion, and promotes apoptosis [177]. 

In another study, azido-peptides were grafted onto GO flakes and subsequent peptide assembly led to the quenching of the nanomaterial’s inherent fluorescence. The peptide sequence was designed to display a target motif that could be cleaved by matrix metalloproteinases (MMP-2). When tested in cell culture, these enzymes secreted by cells could be detected and quantified, since their enzymatic activity led to the disassembly of the nanomaterial and to the consequent switching-on of the fluorescent signal [137]. A similar concept was also developed for thrombin biosensing. In this case, GO flakes were decorated with gold nanoparticles and self-assembling peptide-displaying cleavable sites for the enzyme. As a result, in the presence of thrombin, the absorbance of the nanomaterial varied and allowed for the spectroscopic detection of the enzyme [133].

#### 2.4.2. Energy-Harvesting Systems for Bioelectronics

**Figure 9:**
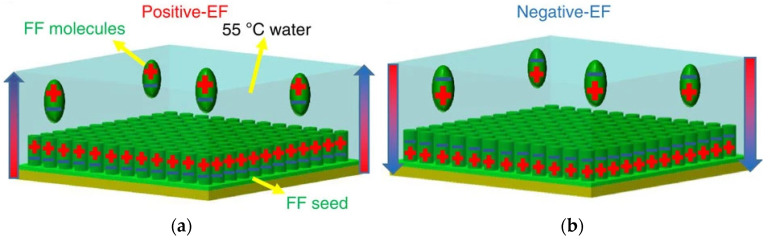
Diphenylalanine (FF) self-assembly into vertically aligned nanotubes can be attained with controlled polarization upon application of a positive (**a**) or negative (**b**) electric field (EF), to construct piezoelectric devices for bioelectronics. Adapted from [178] under a Creative Commons license (https://creativecommons.org/licenses/by/4.0/, accessed on 8 June 2021).

The piezoelectric activity of diphenylalanine nanotubes has been exploited also to develop energy-harvesting systems for bioelectronics to generate charge when pressed. To this end, they are usually grown vertically aligned through evaporation of a saturated solution with a seed of molecules onto a substrate, and controlled polarization of the peptides can be attained by applying an electric field (Figure 9) [178]. Moreover, horizontal alignment onto GO flexible substrates can be achieved, thanks to a difference in wettability and an applied electrical field [179].

#### 2.4.3. Biomaterials for Wound Healing and Tissue Regeneration

There is growing interest in the conjugation of antimicrobial peptides with antibacterial graphene-based materials [180], for instance for potential applications in wound healing. To this end, one-pot ring-opening copolymerization of lysine and leucine *N*-carboxyanhydride into self-assembling polypeptides was used as a simple strategy to yield self-assembling antimicrobials that were also conjugated to graphene to yield hierarchical structures [181]. Antibacterial membranes were developed by combining a self-assembling palmitoyl tetrapeptide with GO flakes [134]. Self-assembling diphenylalanine was also reported to exert antimicrobial activity through bacterial membrane disruption [182], and was studied in combination with GO flakes for potential applications in nanomedicine [132].

Applications of peptide–graphene supramolecular systems are wide, and they could be envisaged also for the regeneration of conductive tissues thanks to the electronic properties imparted especially by the carbon nanostructure [140]. To this end, reduced graphene oxide flakes were grafted with bioactive peptides to yield layer-by-layer supramolecular scaffolds held together by electrostatic interactions. The scaffolds allowed for enhanced adhesion and neurite outgrowth in PC12 cells cultured with electrical stimulation [141].

This type of material has been envisaged also for intervertebral disc repair, as the GO acted as nanofiller to reinforce the hydrogel formed by the self-assembling FEFKFEFK sequence. Interestingly, at pH = 4 and a low concentration of peptide, electrostatic interactions were attractive and the viscous modulus of the hydrogel was increased. However, at a high concentration of peptide, electrostatic interactions can be absent or repulsive, and the elastic modulus was decreased. At pH = 7, there was an increase in elastic modulus at all concentrations, as hydrophobic interactions dominated over the electrostatic ones, which were removed upon conditioning with culture media [135]. The same peptide was also tested, together with another two sequence variant, for self-assembly in the presence of graphene-based materials as nanofillers to provide scaffolds that were tested for the culture of stem cells. Moreover, in this case, the intimate interaction between peptides and nanocarbon flakes were key determinants for the viscoelastic properties of the final systems, with a positive effect arising from hydrophobic interactions and variable outcomes arising from electrostatic forces [139]. 

Inorganic–organic bio-hybrids have been developed for tissue regeneration too. For instance, the self-assembling peptide AEAKAEAKYWYAFAEAKAEAK was used to provide nanofibers which, combined with GO flakes, favored the nucleation and growth of hydroxyapatite crystals for bone tissue regeneration [136]. Recently, 2D sheets arising from the self-assembly of the LLVFGAKMLPHHGA peptide sequence were combined with GO and hydroxyapatite to yield porous and light-weight scaffolds for the reconstruction of bones [130]. 

Hydrogels obtained from resilin-like polypeptides also displayed enhanced mechanical resilience upon inclusion of GO flakes [183], as did those formed from a heterochiral tripeptide [66]. Alternatively, graphene can be used as a nanofiller for supramolecular hydrogels arising from the self-organization of peptide derivatives, as shown for a pseudopeptide, yielding biomaterials with a thermoresponsive and thixotropic behavior [184].

#### 2.4.4. Drug Release

**Figure 10:**
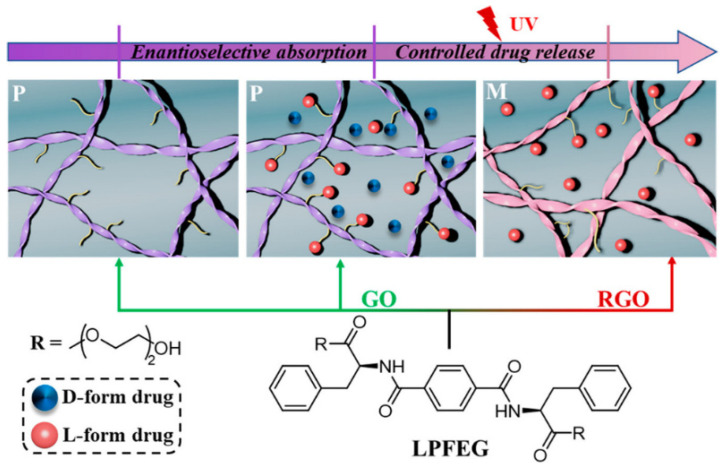
Photo-triggered ibuprofen release from self-assembled helical nanoribbons on the surface of GO flakes. Reprinted with permission from Zhang, Y. et al., *ACS Nano* [172] copyright © 2021 American Chemical Society.

In certain cases, a very interesting supramolecular behavior can arise from the interaction between self-assembling peptides and graphenic surfaces. Racemic crystals from biomolecules, such as self-assembling phenylalanine, were found to maintain a straight geometry when adsorbed onto a flat surface; conversely, enantiopure analogues underwent twisting [124]. This type of phenomenon could be exploited for the enantioselective drug adsorption and release, as shown for ibuprofen and a self-assembling phenylalanine derivative that formed right-handed helical nanoribbons on the surface of GO. UV-irradiation could then be used as a trigger for drug release, following the switching of helicity to left-handed (Figure 10) [185].

## 3. Conclusions

The combination of self-assembling peptides and carbon nanostructures is a research area undergoing significant expansion. The very different chemical nature of these two components represents a technical challenge that requires a wide skillset to efficiently conjugate them in stable systems. However, it also represents an opportunity for a qualitative leap in the biomedical field, if the advantages of each component are put to good use, ideally creating synergy. While in the past the inclusion of carbon nanostructures, and especially graphene, was mainly studied with the aim to simply enhance the mechanical stability and resilience of peptide gels, in recent years a great progress towards more complex applications has been made in the biomedical field.

In particular, the electronic properties of peptide assemblies have attracted great attention also for energy-harvesting and conversion, leading to innovative approaches in the field of bioelectronics and biosensing where carbon nanostructures are already well-known for their excellent performance. The vast majority of these studies focus on the simple diphenylalanine building block, but it is anticipated that extending the chemical diversity of peptide sequences will allow to better benefit from these biomolecules and their potential (bio)activities. However, a challenge lies in the identification of general trends to predict the physicochemical properties and supramolecular behavior of a large variety of self-assembling peptides. This class of biomolecules is well-known for its great chemical diversity which, combined with the inherent flexibility of biomolecules, renders prediction of their behavior particularly challenging. We anticipate that machine learning methods will become key for the de novo design of self-assembling peptides that favorably interact with carbon nanomaterials, thanks to the increasing growth of large datasets that are being generated for these compounds worldwide.

It is clear that, thus far, graphene-based materials represent the most studied nanocarbon type to develop systems that interface them with self-assembling peptides, followed by carbon nanotubes, and, more recently, carbon dots. This latter type of nanocarbon is clearly on an ascending trajectory for future applications, thanks to the small-size, tunable luminescence, and possibility for green synthesis, all of which make it highly promising for biomedical applications. Considering the emerging fluorescent properties of amyloids [14,186], one can envisage further opportunities to innovate in the biomedical field from the optical applications that combine these two components into multifunctional theranostic systems for multimodal imaging. Clearly this type of application is particularly challenging to develop as it requires both interdisciplinary and multidisciplinary skills, spanning from chemistry to engineering and biology. Therefore, financial and networking support is vital to sustain the free exchange of knowledge between scientists of different backgrounds that approach this exciting area of research, to allow for progress that will benefit the society as a whole. 

## Figures and Tables

**Table 1:** Recent examples of studies on the interaction between self-assembling peptides and carbon nanostructures.

Carbon Nanostructure	Peptide	Material	Application	Ref.
Fullerene	Aβ(1—40), Aβ(1—42)	Solution	Amyloidosis inhibition	[112]
Fullerene	Aβ(1—42)	Solution	Amyloidosis inhibition	[113]
Fullerene	Cyclopeptide nanotube	Solution	Bioelectronics	[114]
Fullerene	Fmoc-Phe-Phe	Hydrogel	Antibacterials	[115]
Carbon dots	D-Leu-L-Phe-L-Phe	Hydrogel	Biomaterial, biosensing	[116]
Carbon dots	Aβ(1—42)	Solution	Amyloidosis inhibition	[117,118]
Carbon dots	Aβ(1—42)	Solution	Biosensing	[119]
Carbon dots	Biofilm amyloids	Solution	Antibacterials	[120]
Carbon dots	RGDAEAKAEAKYWYAFAEAKAEAKRGD	Solution	Theranostics	[121]
Carbon dots, GO ^1^	AEAKAEAKYWYAFAEAKAEAK	Solution	Biosensing	[111]
Carbon dots, CNTs, GO ^1^	Aβ_33—42_	Solution	Amyloidosis inhibition	[67]
CNHs, CNTs, GO ^1^	L-Leu-D-Phe-D-Phe	Hydrogel	Biomaterial, drug delivery	[66]
CNTs	EFK8	Hydrogel	Tissue regeneration	[98]
CNTs	RADA16-I	Hydrogel	Tissue regeneration	[122]
CNTs	Boc-β^3^(R)Phe-β^3^(R)Phe-OH Boc-γ^4^(R)-Phe-γ^4^(R)Phe-OH	Fibrils	Tissue regeneration	[123]
CNTs, graphene	Trp, Phe	Solution	Bioelectronics	[124]
CNTs, GO ^1^	C_8_H_16_(-CH_2_-NH-Gly_5_)_2_·2HCl	Film	Tissue regeneration	[100]
CNTs, GO ^1^	Fmoc-Tyr-OH, Fmoc-Tyr(Bzl)-OH	Hydrogel	Drug delivery	[125]
Graphene	Phe-Phe	Solution	Biosensing	[126]
Graphene	IMVTESSDYSSY	Film	Biosensing	[127]
Graphene	HSSYWYAFNNKT IMVTESSDYSSY	Film	Biosensing	[128]
Graphene	YGAGAGAY, EGAGAGAE, RGAGAGAR	Solution	Biosensing	[129]
Graphene	LLVFGAKMLPHHGA	Scaffold	Tissue regeneration	[130]
GO ^1^	Phe-Phe	Solution	Biosensing	[131]
GO ^1^	Phe-Phe, Tyr-Tyr	Solution	Theranostics	[132]
GO ^1^	CLVPRGSC, CRGC	Solution	Biosensing	[133]
GO ^1^	C_16_CO-KKFF	Membrane	Antibacterials	[134]
GO ^1^	FEFKFEFK	Hydrogel	Tissue regeneration	[135]
GO ^1^	AEAKAEAKYWYAFAEAKAEAK	Solution	Tissue regeneration	[136]
GO ^1^	N_3_-KKPPPPKGPLGVRGC-CONH_2_ N_3_-KKPPPPKGPLGVRGA-CONH_2_ N_3_-KKPPPPKAAPFC-CONH_2_	Solution	Biosensing	[137]
GO ^1^	Block copolymer polypeptide PBLG-b-PDMS-b-PBLG	Gel	Tissue regeneration	[138]
GO ^1^, rGO ^2^	VEVKVEVK, FEFKFEFK, FEFEFKFE	Hydrogel	Tissue regeneration	[139]
rGO ^2^	Boc-Trp-PABA-OMe, Boc-Phe-PABA-OMe, Boc-Phg-PABA-OMe	Hydrogel	Tissue regeneration	[140]
rGO ^2^	Polylysine, polyglutamate	Dispersion	Tissue regeneration	[141]

^1^ GO—graphene oxide. ^2^ rGO—reduced graphene oxide.
